# Neutrophil CD64 expression levels in IGRA-positive individuals distinguish latent tuberculosis from active disease

**DOI:** 10.1590/0074-02760180579

**Published:** 2019-04-08

**Authors:** Raquel da Silva Corrêa, Luciana Silva Rodrigues, Leticia Hagge Lima Pereira, Otto Castro Nogueira, Janaína Leung, Marcela da S Sousa, Mariana de Andrea Hacker, Helio Ribeiro de Siqueira, Domênico Capone, Rogério Lopes Rufino Alves, Maria Cristina Vidal Pessolani, Veronica Schmitz, Geraldo Moura Batista Pereira

**Affiliations:** 1 Universidade do Estado do Rio de Janeiro, Faculdade de Ciências Médicas, Laboratório de Imunopatologia, Rio de Janeiro, RJ, Brasil; 2 Fundação Oswaldo Cruz-Fiocruz, Instituto Oswaldo Cruz, Laboratório de Microbiologia Celular, Rio de Janeiro, RJ, Brasil; 3 Universidade do Estado do Rio de Janeiro, Hospital Universitário Pedro Ernesto, Serviço de Pneumologia e Tisiologia, Rio de Janeiro, RJ, Brasil; 4 Fundação Oswaldo Cruz-Fiocruz, Instituto Oswaldo Cruz, Laboratório de Hanseníase, Rio de Janeiro, RJ, Brasil

**Keywords:** tuberculosis, latent tuberculosis infection, biomarkers, neutrophils, CD64

## Abstract

**BACKGROUND:**

CD64 (FcγR1) is a high-affinity receptor for monomeric IgG1 and IgG3. Circulating neutrophils express very low amounts of CD64 on their surface.

**OBJECTIVES:**

Our primary aim was to investigate the utility of neutrophil CD64 surface expression as a biomarker of active pulmonary tuberculosis (TB). We hypothesised that elevated neutrophil CD64 expression in TB infection would be associated with interferon gamma (IFN-γ) as an inducer of CD64 expression.

**METHODS:**

The expression level of CD64 per neutrophil (PMN CD64 index) was quantitatively measured with flow cytometry using a Leuko64 kit in samples from patients with TB and latent TB infection (LTBI) as well as healthy controls, as part of a prospective cohort study in Brazil.

**FINDINGS:**

The PMN CD64 index in patients with TB was higher than that in healthy controls and LTBI. Receiver operating characteristic curve analyses determined that the PMN CD64 index could discriminate patients with TB from those with LTBI and healthy individuals. PMN CD64 index levels returned to baseline levels after treatment.

**CONCLUSIONS:**

The positive regulation of CD64 expression in circulating neutrophils of patients with active TB could represent an additional biomarker for diagnosis of active TB and could be used for monitoring individuals with LTBI before progression of TB disease.

Tuberculosis (TB) remains a major global health problem, with an estimated 10.4 million incident cases and 1.3 million TB deaths in 2016.^1^ In most people, the initial *Mycobacterium tuberculosis* (MTB) infection is eliminated or controlled by the host’s defences. Latent TB infection (LTBI) is defined as a state of persistent immune response to *M. tuberculosis* without clinically-manifested evidence of active TB disease.^2^ However, LTBI may evolve to active disease, perpetuating the transmission chain, morbidity, and mortality. The rapid identification and suitable treatment (chemoprophylaxis) of LTBI are therefore critical for reducing the risk of development of disease and death caused by TB, to achieve the goals of the End TB Strategy.^3^

Current tests for identification/diagnosis of LTBI include the interferon gamma release assay (IGRA) and tuberculin skin test (TST). Both tests are immunity based and have limited ability to predict disease or to identify individuals in whom the TB infection is likely to progress to active disease. In addition, TST has cross-reactivity with environmental mycobacteria and *M. bovis* bacillus Calmette-Guérin (BCG) vaccination.^4^ In addition to TST and IGRA, C-Tb is another test for the diagnosis of LTBI.^5^ C-Tb is applied in the same way as the TST but rather than using a purified protein derivative tuberculin antigen, the C-Tb uses the recombinant proteins ESAT-6 and CFP-10. C-Tb has very high concordance with IGRA and similar specificity in negative controls. In contrast to the TST, previous BCG vaccination does not compromise the specificity of C-Tb.^6^ Given the importance of TB for global health and the limitations of the current tests, it would be very useful to have a new, simple, rapid diagnostic test to screen patients for LTBI and to predict active TB.

Transcriptional profiling provides insights into biomarkers and networks that may provide new molecular targets of infectious and non-infectious diseases.^7^ Berry et al. reported that using microarray analysis, the TB signature is controlled by a neutrophil-driven interferon (IFN)-inducible gene pattern, consisting of both IFN-γ and type I IFN-αβ signalling, which correlates with lung radiographic disease severity.^8^ In particular, circulating neutrophils of patients with active TB show an increase in programmed death ligand-1 expression, which is reduced with anti-mycobacterial chemotherapy.^9^ These study findings support a role for neutrophils in the pathogenesis of TB.

Gene expression profiles of peripheral blood mononuclear cells from patients with TB and individuals with LTBI reveal that lactoferrin, FCGR1A (high-affinity IgG Fc receptor 1 (CD64)), and Ras-associated GTPase 33A are sufficient for the classification of (i) patients with active TB, (ii) LTBI, and (iii) noninfected healthy donors.^10^ In addition, monocyte CD64 cell surface expression is higher in patients with TB than among healthy *M. tuberculosis*-infected donors;^10^ neutrophils were not included in the above analysis. Considering that IFN-γ is an inducer of CD64 expression on neutrophils,^11^ we sought to define the neutrophil CD64 expression using flow cytometry analyses, to detect the different phases of *M. tuberculosis* infection (LTBI, active TB) and investigate the use of this approach as an additional biomarker of TB.

## SUBJECTS AND METHODS

*Study design and participants* - A total of 53 individuals were prospectively enrolled in this study: 16 healthy donors (HDs), 14 individuals with latent *M. tuberculosis* infection (LTBI), and 23 individuals diagnosed with pulmonary TB. The HD group included healthy adults without any signs or symptoms of active pulmonary TB and with negative test results for IGRA and IFN-γ-inducible protein-10 (IP-10). The case definition of LTBI was healthy adults with no signs or symptoms of active pulmonary TB but with positive IGRA and/or IP-10 test results. TB was diagnosed based on clinical, radiological, microbiological, and pathological criteria. All HDs and participants with LTBI had normal chest radiography results. Individuals with a previous history of TB, human immunodeficiency virus (HIV) infection, silicosis, end-stage renal disease, leukaemia/lymphoma, positive TST in the past 16 months, or previous anti-TB or immunosuppressive therapy for more than two weeks were excluded from the study. Pregnant women were also excluded. TB was confirmed by the presence of acid-fast bacilli on microscopy using the Ziehl-Neelsen method and/or identification of *M. tuberculosis* in a sputum culture. Patients with negative sputum smear and culture results but with clinical and radiographic findings compatible with active pulmonary TB were classified as having clinical TB. All patients were treated in accordance with the Brazilian Ministry of Health guidelines. Treatment was provided free of charge by the National Tuberculosis Control Program and administered under direct supervision of qualified health care workers.

*QFT-IT antigen-specific IFN-*g *assay* - The IFN-γ release assay was performed using QuantiFERON-TB Gold in-Tube (QFT-IT; QIAGEN, Valencia, CA, USA) kits, according to the manufacturer’s instructions. Briefly, 1 mL of whole blood was drawn into three QFT-IT tubes precoated with saline (Nil, negative control), MTB-specific antigens (ESAT-6, CFP-10, and TB 7.7), or mitogen (positive control) and incubated for 24 h at 37ºC. After centrifugation, the supernatant was collected and stored frozen at -20ºC until cytokine quantification using enzyme-linked immunosorbent assay (ELISA). IFN-γ concentrations were determined using a QFT Gold kit (QIAGEN). The results were calculated using the manufacturer’s QFT-GIT software (version 2.6). A positive result was defined as [(MTB antigen-stimulated IFN-γ level)-(Nil-stimulated IFN-γ level)] ≥ 0.35 IU/mL.

*QFT-IT antigen-specific IP-10 assay* - Levels of IP-10 in the IGRA supernatant were quantified using the same supernatant as for IFN-γ from the QFT-IT tubes, using a commercially available ELISA kit (Duoset; R&D Systems, Minneapolis, MN, USA). The detection limit of this kit is 31.25 pg/mL. ELISA was done according to the manufacturer’s instructions. All assays were performed in duplicate. Results were expressed in pg/mL after processing the data using SoftMax Pro software, version 4.8 (Molecular Devices, LLC, San Jose, CA, USA). We determined the area under the curve (AUC) and cut-off values. Significant AUC analysis results were obtained [AUC, 0.8750; 95% confidence interval (CI), 0.744-1.006, p < 0.0001] for the IP-10 response to ESAT-6, CFP-10, and TB 7.7. For scoring purposes, we chose a cut-off point to maximise the sum of sensitivity and specificity. A positive result was defined as [(MTB antigen-stimulated IP-10 level) ― (Nil-stimulated IP-10 level)] ≥ 535.9 pg/mL.

*Cell staining and flow cytometry analysis* - Expression of CD64 was determined using blood samples, as previously described.^12^ A Leuko64 test kit was purchased from Trillium Diagnostic (Bangor, ME, USA) and sample acquisition was done via a FACSCalibur flow cytometer (Becton Dickinson, NY, USA). Samples were analysed using QuantiCALC software (Trillium Diagnostics). Leukocytes were identified based on their logarithmic side scatter dot-plot profiles. CD163 antibody was included in the kit to differentiate neutrophils from monocytes. A gate was set around the different cell populations and mean fluorescent intensity (MFI) was defined as the geometric mean of the logarithmic fluorescence intensities emitted by the respective leukocyte subset. CD64 and CD163 indices were calculated according to the ratio of MFI of the cell population to that of the beads. In addition, an internal negative control (lymphocyte leuko64 index < 1) and an internal positive control (monocyte leuko64 index > 3) were used to validate each sample.^13^

*Statistics* - Statistical analysis was performed using GraphPad PRISM version 6 (GraphPad Software, San Diego, CA, USA). The data were not normally distributed and comparisons between the four groups of variables were examined using the Kruskal-Wallis test with Dunn’s multiple comparison post-test. The adopted statistical significance level was 0.05 with power of 80%. Sample size for evaluating the PMN CD64 index was calculated using OpenEpi software.^14^ The sample size required per group was six individuals, considering the number of groups, with a maximum mean difference of 2 and an expected standard deviation of 1.5. The optimal cut-off value for the PMN CD64 index was determined using receiver operating characteristic (ROC) curve analysis.

*Ethics* - Written informed consent was obtained from all individuals included in the study. Participants up to 18 years of age were recruited at the Pulmonology and Tisiology Service, Pedro Ernesto University Hospital/Rio de Janeiro State University (HUPE/UERJ), Rio de Janeiro state, Brazil. The protocol for this study was reviewed and approved by the Institutional Ethics Committee (SISNEP registry no. 2612/2010).

## RESULTS

*Characteristics of the study population* - We enrolled 30 participants who had no symptoms of TB. First, blood was drawn for detection of IFN-γ and IP-10 using the QFT-IT tubes. Additionally, participants underwent a TST. QFT-IT testing was planned for all participants before skin test agents were administered, to avoid possible booster responses. The groups were defined based on the results for IFN-γ and IP-10. Eighteen participants (33.9%) were TST positive (> 5mm). Four participants had TST^5-10mm^ and 14 had TST^>10mm^; of the latter, 4 had TST^³15 mm^. The remaining 15 participants were TST negative; 15 (28.3%) had TST^0mm^, and none (0%) had TST^1-4mm^. Most participants were male (60.4%), and 81.4% had at least one BCG scar. We also enrolled 15 participants with active pulmonary TB before treatment and eight after treatment. Table summarises the demographic, clinical, and laboratory characteristics of the 53 participants.

**TABLE Characteristics:** of the enrolled individuals

Enrolled subjects (%)	HD	LTBI	Active TB	Cured TB	Total
	16 (30.2)	14 (26.4)	15 (28.3)	8 (15.1)	53 (100)
Median age	33	46	47	40.5	
Range	22-55	32-63	23-74	20-80	
Male gender (%)	5 (31.2)	8 (57.1)	10 (66.7)	4 (50)	33 (47.1)
					
BCG scar (%)					
Yes	16 (100)	12 (85.7)	8 (53.3)	6 (75)	59 (81.4)
No	0 (0)	2 (14.3)	6 (46.7)	2 (25)	12(18.6)
					
TST					
Mean ± SE	5.0 ± 1.7	10.0 ± 1.9	8.7 ± 5.2		
Positive (%)	6 (37.5)	10 (71.4)	2 (13.3)		
Negative (%)	10 (62.5)	4 (28.6)	1 (6.7)		
ND	0 (0)	0 (0)	12 (80)		
					
IGRA Test					
Positive (%)	0 (0)	18 (75)	6 (40)		
Negative (%)	23 (100)	6 (25)	7 (46.7)		
ND (%)	0 (0)	0(0)	2 (13.3)		
IP-10Test					
Positive (%)	0 (0)	21 (87.5)	7 (46.7)		
Negative (%)	23 (100)	3 (12.5)	6 (40)		
ND (%)	0	0	2 (13.3)		
					
TB diagnosis (%)					
Culture					
Positive			15 (65.2)		
Negative			1 (4.3)		
Missing			7 (30.4)		
Sputum					
AFB positive (1+)			1 (4.3)		
AFB positive (≥ 2+)			8 (34.7)		
AFB negative			8 (34.7)		
Missing			6 (26.1)		

BCG: Bacillus Calmette et Guérin; HD: healthy donors; LTBI: latent tuberculosis infection; ND: not determined; SE: standard error; TB: tuberculosis; TST: tuberculin skin test.

**Fig. 1:**
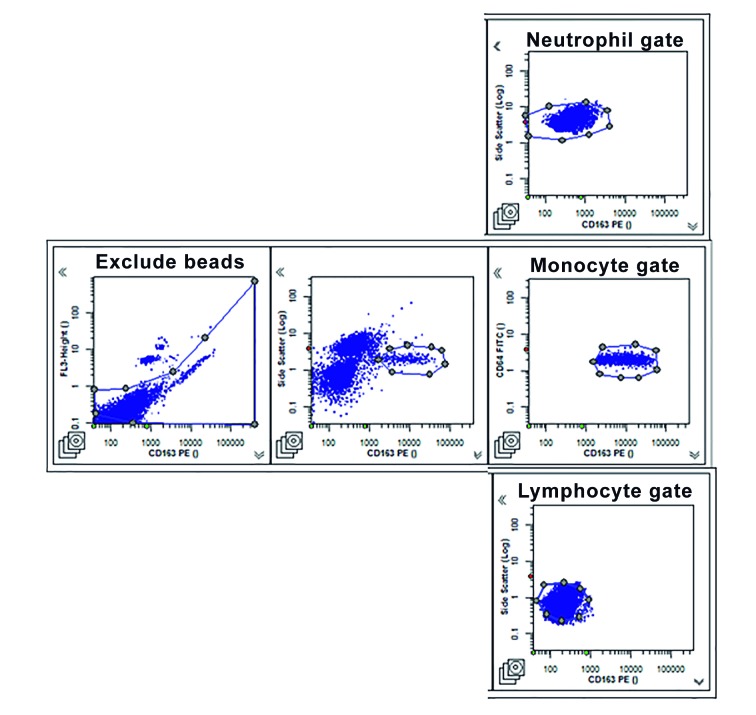
representative gating of neutrophils, monocytes, and lymphocytes in CD64 FITC/CD163 PE FACS diagrams. Panel depicts representative patterns of neutrophil, monocyte, and lymphocyte gates.

**Fig. 2:**
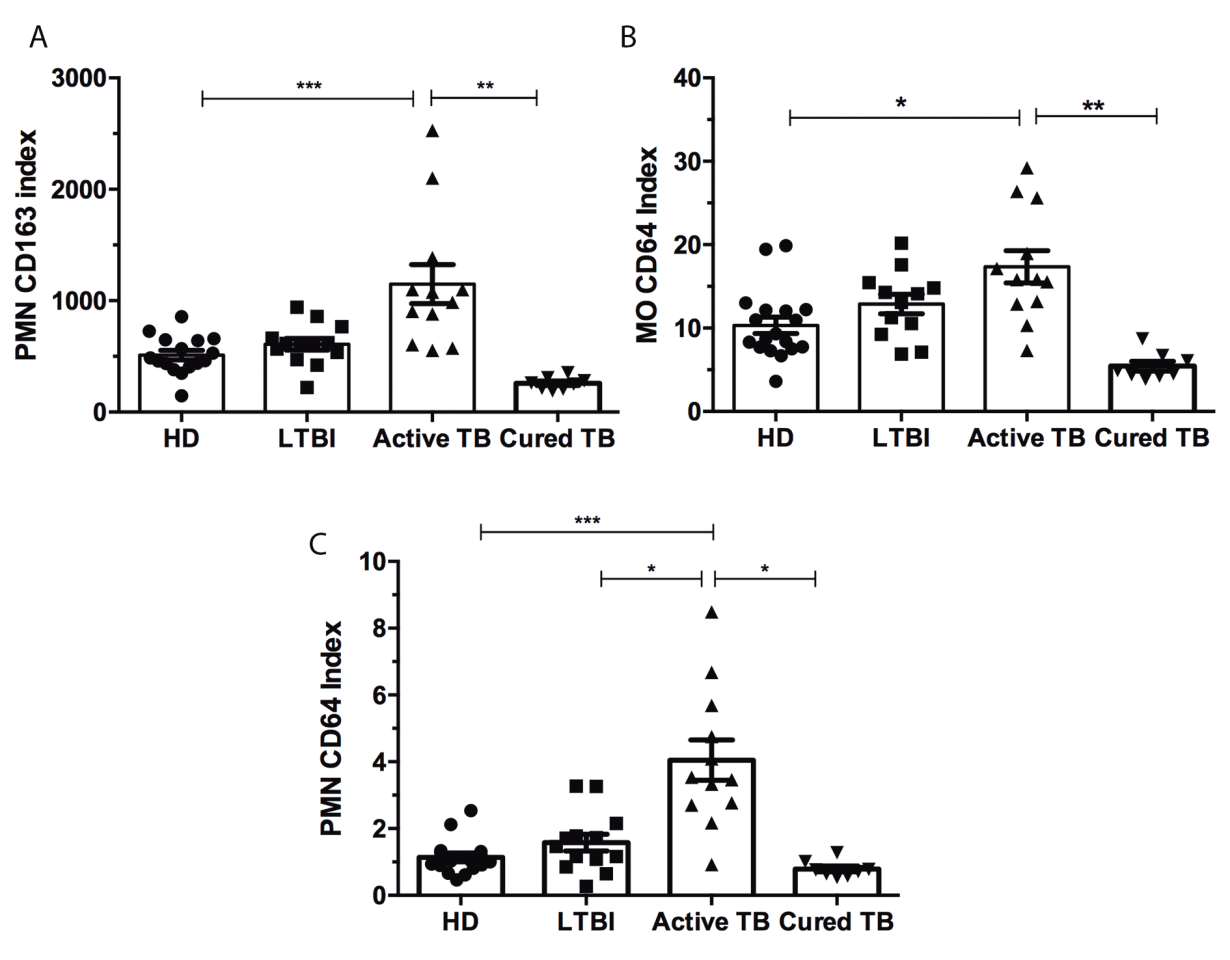
CD64 expression on circulating neutrophils is elevated in patients with active tuberculosis (TB). (A) Cytometry analyses of surface expression of CD163 on neutrophils (PMN CD163 index) from whole blood. (B) Cytometry analyses of CD64 surface expression on circulating monocytes (MO CD64 index) and (C) neutrophils (PMN CD64 index). Each filled dot represents a participant: healthy donor (HD, n = 16), patient with latent TB infection (LTBI, n = 14), patient with active TB (n = 15), or patient with cured TB (n = 8). Bars represent median ± SEM. Kruskal-Wallis test (* p < 0.05, ** p < 0.01, and *** p < 0.001).

*High neutrophil CD64 expression is associated with active TB disease* - Surface expression of CD64 on circulating blood cells was determined by flow cytometry using the Leuko64 commercial kit. CD64 expression was determined in whole blood samples of patients with TB (n = 15) and LTBI (n = 14) as well as HDs (n = 16). The gate definition of neutrophils and monocytes was determined with staining for CD163, a monocyte-specific antigen that increases the specificity of leukocyte subpopulation identification, associated with side scatter parameters^15^ (Fig. 1). CD64 and CD163 surface expression are presented as an index for each cell population. Monocyte CD163 indices were not different among the groups (data not shown). However, neutrophil CD163 index values in patients with active TB were about 2.2-fold higher than those among HDs (1149 ± 174.6 and 512 ± 42.4, respectively; p < 0.01) (Fig. 2A). Comparative analyses of patients with TB (n = 8) before and after treatment was performed and the results demonstrated that neutrophil CD163 expression dropped about 4.4-fold after treatment (1149 ± 174.6 and 258.2 ± 19.6, respectively; p < 0.01) (Fig. 2A).

Monocyte CD64 index values in patients with active TB were about 1.6-fold higher than those among HDs (17.4 ± 1.9 and 10.3 ± 1.0, respectively; p < 0.05) (Fig. 2B). There were no significant differences detected in the monocyte CD64 index between patients with LTBI and active TB disease (Fig. 2B). TB treatment decreased CD64 expression on monocytes about 3.2-fold (17.4 ± 1.9 and 5.4 ± 1.6, respectively; p < 0.001) (Fig. 2B).

To elucidate whether over-representation of CD64 in the blood of patients with TB^10^ results from increased expression by neutrophils in addition to monocytes, we gated the neutrophil population, performed the analyses, and compared the groups. As shown in Fig. 2C, median values of the PMN CD64 index in patients with TB were about 3.2-fold higher than those among HDs (3.619 ± 0.5429 and 1.134 ± 0.1336, respectively; p < 0.0001) and about 2.3-fold higher than those among participants with LTBI (3.619 ± 0.5429 and 1.579 ± 0.2508, respectively; p < 0.01). Conversely, no significant differences were observed among HDs and participants with LTBI (Fig. 2C). Importantly, the elevated PMN CD64 index in patients with pulmonary TB returned to background levels (0.8467 ± 0.1063; n = 8) following successful anti-TB chemotherapy (Fig. 2C). We next examined whether INF-g production is associated with increased neutrophil CD64 expression in patients with TB. The quantitative expression of CD64 showed a correlation with IGRA results (r = 0.4788; p = 0.0012).

**Fig. 3:**
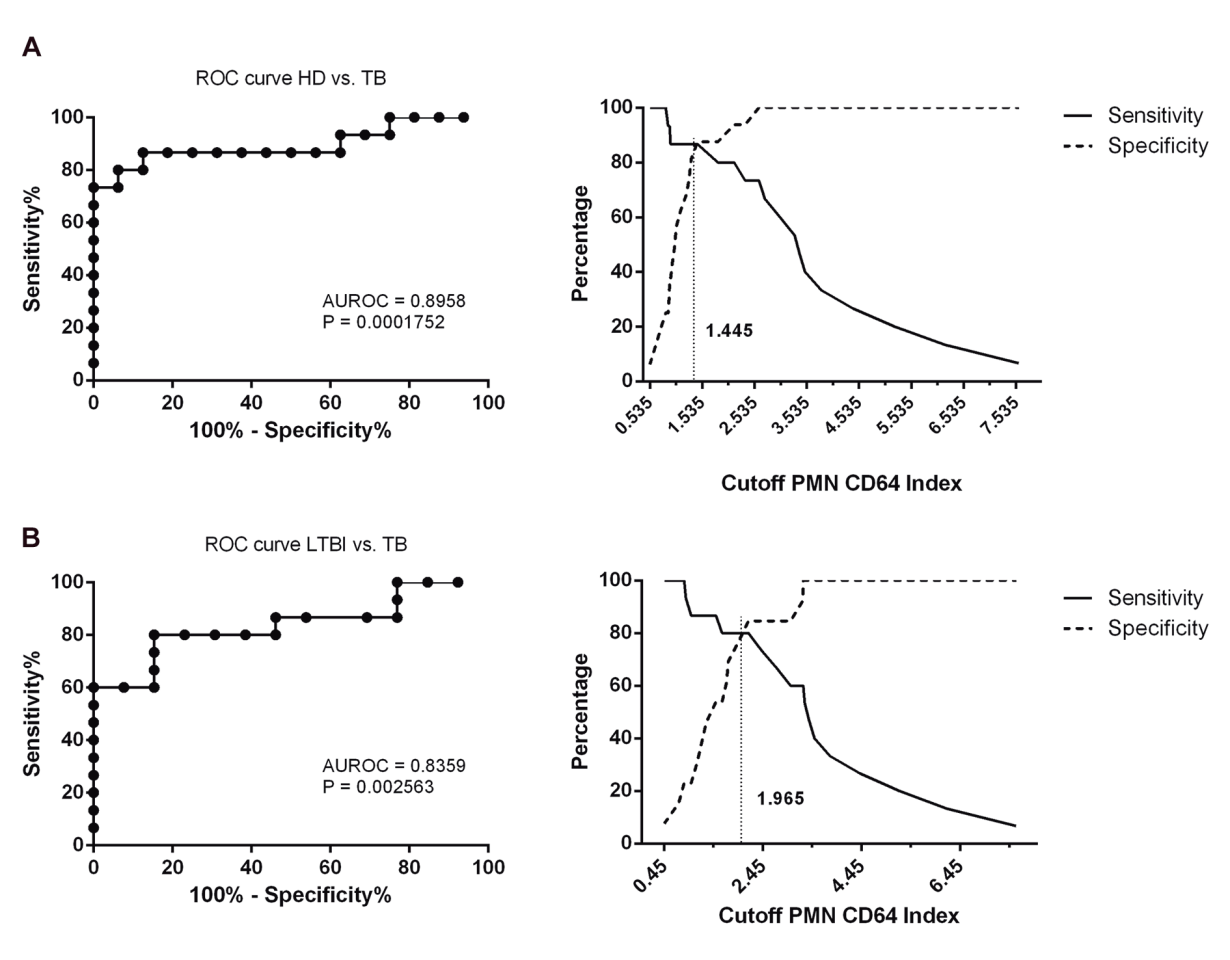
receiver operating characteristic (ROC) curves for the PMN CD64 index. (A) ROC curves comparing the PMN CD64 index in patients with active tuberculosis (TB) versus healthy donors (HDs). (B) ROC curves comparing the PMN CD64 index from patients with latent TB infection (LTBI) versus active TB.

*Accuracy of neutrophil CD64 expression* - ROC curves were constructed and AUC analyses highlighted the utility of the PMN CD64 index for distinguishing between patients with TB and either HDs (AUC, 0.8958; 95% CI, 0.7705-1.021, p = 0.0001752; Fig. 3A) or participants with LTBI (AUC, 0.8359; 95% CI, 0.6818-0.9900, p = 0.002563; Fig. 3B). According to ROC curve analysis, the cut-off points with the best sensitivity and specificity of the PMN CD64 index in distinguishing between TB and either HDs or LTBI were 1.445 and 1.965, respectively (Fig. 3A-B). Interestingly, evaluation of the PMN CD64 index led to the highest accuracy for discriminating active TB from LTBI or HDs, with a sensitivity of 86.67% and 80.00%, and specificity of 87.50% and 76.96%, respectively.

## DISCUSSION

Currently, there is no clear biomarker for distinguishing LTBI from active TB. Additional biomarkers for the diagnosis of both LTBI and active TB disease are urgently needed to improve the available diagnostic tools. The distinction of disease status, assessment of the risk of progression to active disease, and monitoring of successful treatment are critical bottlenecks in the development of new molecular targets. Neutrophil CD64 expression has been reported during the course of several inflammatory disorders.^16^ In this study, we demonstrated that active pulmonary TB induces robust expression of CD64 on neutrophils detected in whole blood, suggesting its potential use as a biomarker for TB. We found that the subset of neutrophil CD64 has greater diagnostic accuracy for distinguishing active TB from LTBI, with sensitivity for active TB diagnosis of 80.00% and specificity of 76.92%. Moreover, we were able to detect a reduction in neutrophil CD64 levels observed in patients following chemotherapy.

CD64, a high-affinity type I immunoglobulin Fc receptor (FcγR1) that recognises IgG1 and lgG3, is constitutively expressed in monocytes. Neutrophil CD64 expression is negligible in the healthy state^17^ and is directly upregulated by IFN-γ *in vitro* and *in vivo*.^11^ Increased expression of CD64 on neutrophils is observed in certain bacterial infections and is an early biomarker of severity and outcome in sepsis.^16^ ^,^ ^18^ CD64 expression on neutrophils remains effective for detection of infection in patients with rheumatoid arthritis, even in those using biologic response modifiers or corticosteroids.^19^ ^,^ ^20^ Recently, we reported that surface CD64 expression on circulating neutrophils increased significantly in erythema nodosum leprosum, an immunological complication of multibacillary leprosy.^12^ The higher levels of CD64 on circulating neutrophils were correlated with disease severity. However, neutrophil CD64 expression is not a good parameter for detecting *M. leprae* infection as it does not increase in patients with non-reactional forms of the disease.^12^

Our data reveal that there is an increase in the level of CD163 expression on neutrophils in patients with active TB. CD163, a haemoglobin scavenger receptor, is exclusively expressed by the monocyte-macrophage lineage.^15^ Previous studies have indicated that high expression of CD163 is associated with type-2 macrophages^21^ and is a marker of an anti-inflammatory and tissue homeostatic macrophage subclass.^22^ CD163 has been previously identified as an indicator of disease severity in several inflammatory and infectious diseases.^23^ ^,^ ^24^ ^,^ ^25^ ^,^ ^26^ High levels of soluble CD163 are associated with increased mortality in TB and serum CD163 has been proposed as a useful prognostic tool in TB.^27^ Although our data indicate that the PMN CD163 index was much lower for neutrophils than for monocytes in active TB, the expression of CD163 on neutrophils could be used to differentiate patients with TB and HDs. Neutrophils expressing CD163 may be related to differentiation of these cells toward an anti-inflammatory N2 subtype, as recently described.^28^

In this work, we evaluated neutrophil CD64 expression as a biomarker for assessing active TB, using a commercially available kit. Flow cytometry quantitative techniques have been applied in most studies researching CD64 expression in myeloid cells.^29^ ^,^ ^30^ This method is based on the gate selection of neutrophils and monocytes in whole blood samples. The Leuko64 kit reports leukocyte expression of CD64 and CD163 as an index using fluorescein-labelled calibration beads. Notably, the assay can be performed in less than 30 min, thereby meeting the needs for clinical practice. We are aware that, however valuable the results, the main limitation of our study is the number of patients under consideration. Further studies are needed to validate the use of neutrophil CD64 in clinical practice as a complementary diagnostic tool for the management of *M. tuberculosis* infection.

The recent development of microfluidic chips for detecting CD64 on neutrophils offers the possibility of a point-of-care test incorporating this parameter.^30^ The use of CD64 detection in neutrophils with second generation skin tests for TB,^6^ or IFN-γ/IP-10 response to *M. tuberculosis* antigens in whole-blood short-term cultures, can be combined as detection methods to provide evidence of active inflammatory disease and exposure to *M. tuberculosis* when evaluating a patient for TB, using only commercial tools that are currently or will soon be available.
